# The predictors of coronary slow flow in patients undergoing coronary angiography

**DOI:** 10.1186/s43044-024-00536-9

**Published:** 2024-08-13

**Authors:** Romi Ermawan, Yusra Pintaningrum, Yanna Indrayana

**Affiliations:** Faculty of Medicine, Mataram University, FK UNRAM, Jl. Pendidikan, No. 37, Mataram, NTB Indonesia

**Keywords:** Coronary angiography, Coronary slow flow, Predictors

## Abstract

**Background:**

A new challenge in coronary artery disease treatment has emerged, where specific populations exhibit ischemic symptoms without any obstruction in the epicardial coronary artery. Instead, they exhibit slow coronary contrast flow, referred to as coronary slow flow (CSF). This study aims to identify several predictors of CSF.

**Results:**

This case–control study was conducted at the Regional General Hospital of West Nusa Tenggara Province in Indonesia from December 2016 to February 2024. The study involved sixty subjects, with 30 in each group of CSF and normal epicardial coronary artery angiogram (NECA). CSF is enforced by the TIMI frame count (TFC) greater than 27 frames. Among all the predictors studied, coronary artery diameter (p < 0.001) and random blood sugar (p = 0.049) were found to affect the CSF significantly. In the multivariate analysis, coronary artery diameter remained a significant predictor (adjusted OR 10.08, 95% CI 2.64–38.50, p < 0.001), with an optimal cut-off point of more than 3.56 mm, a sensitivity of 76.7%, and a specificity of 70.7% (AUC = 0.787, p < 0.001).

**Conclusion:**

The coronary artery diameter strongly predicts CSF in patients undergoing coronary angiography.

## Background

Coronary artery disease (CAD) continues to be a major contributor to mortality and morbidity worldwide, with a high prevalence rate ranging from 5 to 8% [1]. A new challenge has emerged, where specific populations exhibit ischemic symptoms, but their epicardial coronary angiography results show no obstructive signs. Instead, they exhibit slow coronary contrast flow, referred to as coronary slow flow (CSF) [2]. CSF is rare in routine coronary angiography, with an overall incidence rate ranging from 1 to 7% [3].

Tambe et al. first described the CSF phenomenon in 1972 [4–6]. CSF is considered an independent clinical entity that needs to be diagnosed by excluding other clinical backgrounds like coronary ectasia, coronary stenosis, coronary spasms, structural abnormalities of the heart, cardiac conduction abnormalities, and other diseases that cause rheological disorders or hemodynamic changes [4]. Despite being non-obstructive, CSF can still lead to severe clinical manifestations such as myocardial ischemia, life-threatening arrhythmias, recurrent acute coronary syndrome (ACS), and even sudden cardiac death [7]. While most patients with CSF have a relatively good prognosis, chronic and recurring angina can cause a significant decrease in their quality of life [6].

There is currently no agreement on the cause of CSF. However, the best approach involves addressing microvascular dysfunction, inflammation, abnormalities in blood cell morphology and function, platelet dysfunction, endothelial dysfunction, increased microvascular tone, and metabolic syndrome. Unfortunately, no specific CSF treatment is available currently [4, 6, 8]. It is essential to identify and manage predictors associated with CSF. However, unmasking these predictors has been challenging as studies have produced inconsistent results.

This study aims to identify several predictors of CSF compared to the normal epicardial coronary artery angiogram (NECA) group. The predictors include coronary artery diameter, red cell distribution width (RDW), platelet distribution width (PDW), mean platelet volume (MPV), neutrophil–lymphocyte ratio (NLR), platelet-to-lymphocyte ratio (PLR), body mass index (BMI), random blood sugar (RBS), and serum creatinine levels. There has been limited research on similar lines for the Indonesian population. Therefore, this study is expected to provide a better understanding and guide for handling CSF in the future.

## Methods

### Study design and participants

This study was conducted at the Regional General Hospital of West Nusa Tenggara Province in Indonesia. The study was designed as a case–control study, where the subjects were divided into two groups: the CSF group and the NECA group. The CSF group was selected by identifying all CSF cases from December 2016 to February 2024. On the other hand, the NECA group was chosen as the control, based on age and sex categories that were similar to the CSF group. The study included all patients who were over 18 years of age and had undergone coronary angiography. The main indication for coronary angiography in the subjects was pectoral angina. However, patients with atrial fibrillation (AF), ejection fraction below 50%, coronary anomalies such as myocardial bridging, coronary fistula, and anomaly of the coronary ostium, those who had prior revascularization therapy such as percutaneous coronary intervention (PCI) or coronary artery bypass grafting, and those who had undergone any previous heart surgery were excluded from the study. Both group's medical records and coronary angiography results were analyzed to collect the necessary secondary data such as essential patient information, laboratory findings, electrocardiogram, echocardiogram, and coronary angiogram.

CSF is diagnosed based on the criteria established by Beltrame et al. [9] and Gibson et al. [10]. To be diagnosed with CSF, there must be no obstruction in the epicardial coronary artery and a delayed filling of the contrast agent in a distal coronary artery with a TIMI frame count (TFC) greater than 27 frames. The TFC is calculated by determining the first frame where the contrast agent has filled the entire coronary ostium in an anterograde manner and the last frame where the contrast agent reaches the distal artery. For the left anterior descending artery (LAD), a correction factor is applied by dividing the number of frames among the LAD by 1.7, resulting in the corrected TIMI frame count (cTFC). If the cTFC in LAD or the TFC in other coronary arteries is greater than 27, it is considered CSF. Two experienced interventional cardiologists blinded to the TFC and coronary artery diameter assessment take an average of the measurements.

### Statistical analysis

All data collected were analyzed using the SPSS Statistics version 26 program for Mac (IBM Corp., USA). Numerical data that followed a normal distribution were presented as mean ± standard deviation (SD), while numerical data that did not follow a normal distribution were presented as median (Q1–Q3). The distribution of numerical data was tested using the Shapiro–Wilk test. Categorical data were presented as frequency (n) and percentage (%).

Statistical significance was determined using p values < 0.05. An unpaired T-test was used for numerical comparative analytical tests when the data from all groups followed a normal distribution. On the other hand, the Mann–Whitney test was used when any group had an abnormal data distribution. For categorical comparative analytical tests, the Chi-squared test was used. A logistic regression test was used for multivariate statistical analysis. In this test, independent variables were involved in a bivariate test with p-value < 0.100. Further analysis was carried out for multivariate significant predictors using the receiver operating characteristic (ROC) curve. The sensitivity and specificity of the predictor were determined, and the area under the curve (AUC) was defined. The optimal intersection point in predicting CSF events was determined using this analysis.

## Results

This study involved 30 CSF participants, and it was found that the prevalence of CSF at our center was 0.8%. Additionally, 30 subjects were chosen from NECA as a control group based on age and sex characteristics (Levene's test, p = 0.954). The study participants had a mean age of 51.52 ± 8.02 years, with most being smokers (81.7%). The median BMI of the participants was 26.97 (24.01–30.89). The participants' coronary arteries had a mean TFC of 22.53 ± 7.76, and the average diameter was 3.70 ± 0.59 mm. The largest diameter was seen in the right coronary artery (RCA) (3.86 ± 0.84 mm), and only 15.0% of the participants showed tortuosity. The laboratory results indicated that the median RBS was 100.5 (95.2–120.5) mg/dL, the mean hemoglobin (Hb) was 13.90 ± 1.42 g/dL, the median platelet count was 255,950 (219,500–294,800) /uL, and the median creatinine level was 0.9 (0.8–1.1) mg/dL. The characteristics of the subjects are listed in Table 1.

**Table 1:** The characteristics of the subject

Variables	n (%), mean ± SD, median (Q1-Q3), N = 60
Age (years)	51.52 ± 8.02
Sex	
Males	32 (53.3)
Females	28 (46.7)
Smoker	
Yes	11 (18.3)
No	49 (81.7)
Coronary diameter (mm)	3.70 ± 0.59
LAD (mm)	3.72 ± 0.72
LCx (mm)	3.35 (3.01–3.97)
RCA (mm)	3.86 ± 0.84
Coronary tortuosity	
Yes	9 (15)
No	51 (85)
TFC	22.53 ± 7.76
LAD	20.64 ± 7.68
LCx	25.77 ± 9.68
RCA	19.5 (14.12–26.12)
Hb (g/dL)	13.90 ± 1.42
Platelet (/uL)	255,950 (219,500–294,800)
BMI (kg/m^2^)	26.97 (24.01–30.89)

*BMI* body mass index, *Hb* hemoglobin, *LAD* left anterior descending artery, *LCx* left circumflex artery, *Q* quartile, *RCA* right coronary artery, *TFC* TIMI frame count

In the CSF group, 53.3% of the individuals were men. The left circumflex artery (LCx) was the most commonly affected (83.3%), followed by LAD (50.0%) and RCA (43.3%). This finding corresponds with the mean TFC of LCx, which was the highest (33.08 ± 7.18). Out of the cases, 43.3% involved one vessel, 33.3% involved two vessels, and 23.3% involved three vessels. The characteristics of subjects with CSF are listed in Table 2.

**Table 2:** The characteristics of the subjects with CSF

Variables	n (%), mean ± SD, median (Q1-Q3), N = 30
Sex	
Males	16 (53.3)
Females	14 (46.7)
Coronary involvement	
LAD	15 (50.0)
LCx	25 (83.3)
RCA	13 (43.3)
TFC	
LAD	26.76 (23.82–28.46)
LCx	33.08 ± 7.18
RCA	27.47 ± 8.89
Number of involved vessel	
One vessel	13 (43.3)
Two vessels	10 (33.3)
Three vessels	7 (23.3)

*LAD* left anterior descending artery, *LCx* left circumflex artery, *Q* quartile, *RCA* right coronary artery, *TFC* TIMI frame count

**Fig. 1:**
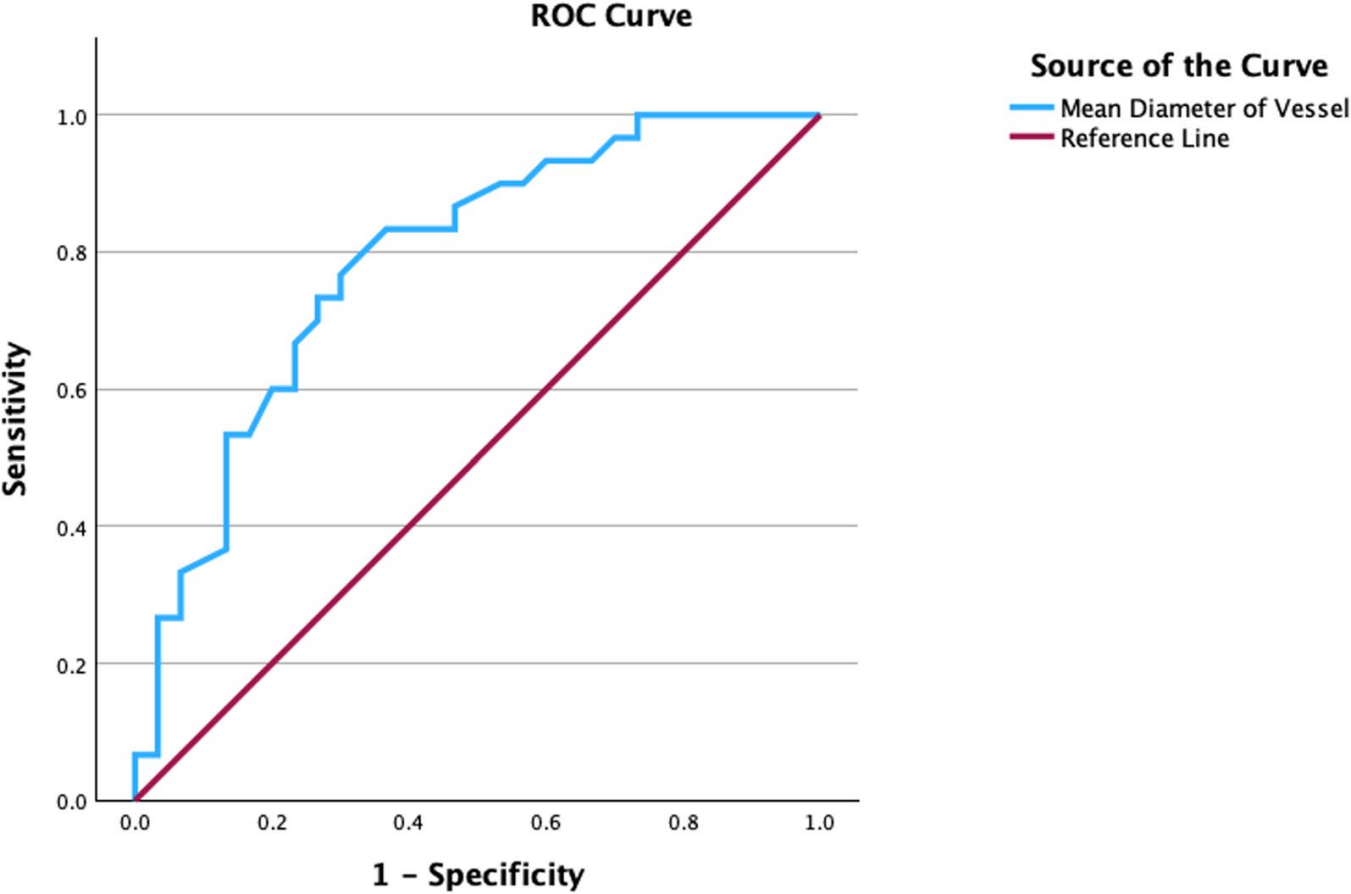
ROC curve analysis of the mean coronary artery diameter in predicting CSF

There was a significant difference in the TFC between the CSF and NECA groups, with values of 29.04 ± 4.52 and 16.02 ± 3.79, respectively (p < 0.001). Only two predictors, coronary artery diameter (3.99 ± 0.53 vs. 3.41 ± 0.51, p < 0.001) and RBS (105.5 (97.0–135.0) vs. 97.5 (95.0–115.0), p = 0.049), were found to affect the CSF significantly. Multivariate logistic regression analysis was conducted on predictors with p < 0.100 values, including coronary artery diameter, RBS, and creatinine levels. It was found that only coronary artery diameter had a significant influence on CSF (adjusted OR 10.08, 95% CI 2.64–38.50, p < 0.001). These findings are listed in Table 3. Furthermore, a ROC curve analysis was performed to determine the difference between the CSF and NECA groups. The optimal cut-off point of the mean coronary artery diameter was more than 3.56 mm, with a sensitivity of 76.7% and specificity of 70.7% (AUC = 0.787, p < 0.001), shown in Fig. 1.

**Table 3:** Effect of several predictors on CSF

Predictors	n (%), mean ± SD, median (Q1-Q3)			Adjusted	
	CSF group, N = 30	NECA group, N = 30	p	OR (95% CI)	p
Age (years)	50.63 ± 8.09	52.40 ± 7.99	0.398^1^		
Smoker					
Yes	7 (63.6)	4 (36.4)	0.506^2^		
No	23 (46.9)	26 (53.1)			
Diameter (mm)	3.99 ± 0.53	3.41 ± 0.51	** < 0.001**^**1**^	10.08 (2.64–38.50)	** < 0.001**^**4**^
RDW (fL)	12.95 (12.10–19.40)	13.25 (12.15–38.15)	0.437^3^		
PDW (fL)	19.60 (12.72–20.72)	19.15 (16.25–20.42)	0.506^3^		
MPV (fL)	8.65 ± 1.76	8.22 ± 1.57	0.330^1^		
NLR	1.83 (1.32–2.61)	1,91 (1.41–2.86)	0.684^3^		
PLR	114.87 (90.34–153.11)	102.98 (84.22–132.74)	0.359^3^		
BMI (kg/m^2^)	27.30 (24.54–31.25)	26.40 (23.23–29.34)	0.344^3^		
RBS (mg/dL)	105.5 (97.0–135.0)	97.5 (95.0–115.0)	**0.049**^**3**^	1.03 (1.00–1.05)	0.066^4^
Creatinine (mg/dL)	1.0 (0.8–1.1)	0.9 (0.7–1.0)	0.080^3^	1.91 (0.16–22.66)	0.608^4^
TFC	29.04 ± 4.52	16.02 ± 3.79	** < 0.001**^**1**^		

*BMI* body mass index, *CI* confidence interval, *CSF* coronary slow flow, *MPV* mean platelet volume, *NECA* normal epicardial coronary artery, *NLR* neutrophil–lymphocyte ratio, *OR* odds ratio, *PDW* platelet distribution width, *PLR* platelet-to-lymphocyte ratio, *RBS* random blood sugar, *RDW* red cell distribution width, *TFC* TIMI frame count

^1^Unpaired t-test

^2^Chi square test

^3^Mann-Whitney

^4^Multivariate logistic regression test

## Discussion

In this study, the prevalence of CSF was found to be 0.8%, which is lower than the prevalence reported by Nakanishi et al. (1–7%) [3]. However, Sanati et al. also published a similar result (< 1%) [5]. The mean age of the subjects with CSF was 50.63 ± 8.09 years, with males accounting for 53.3% of the cases. Several studies have reported that men are more likely to be affected by CSF, as shown by Huang et al. (61.4%) [6] and Yang et al. (63.4%) [11]. Interestingly, this study found that age did not significantly affect the incidence of CSF (p = 0.398), consistent with several other studies [5, 12] showing that age is not a significant predictor of CSF.

Additionally, most of the subjects in this study were non-smokers (81.7%), and smoking was not found to be a significant predictor of CSF (p = 0.506). This finding is not consistent with some other studies that have shown that smoking has a significant effect, such as those conducted by Altun et al. (p = 0.031) [12], Elsanan et al. (adjusted p = 0.006) [13], and Shui et al. (p < 0.001) [14]. However, these discrepancies could be attributed to the small number of smokers in this study, which may have biased the genuine relationship between smoking and CSF.

According to this study, BMI is not a significant predictor of CSF (p = 0.344), which differs from other publications' findings. Sanati et al. (adjusted p = 0.003) [5], Huang et al. (p = 0.010) [6], and Elsanan et al. (adjusted p < 0.001) [13] have published that BMI is a strong predictor of CSF. Increasing BMI has been shown to elevate the risk of cardiovascular mortality by increasing vasoconstriction mediated by the sympathetic nervous system and systemic inflammatory processes [15, 16]. Obese populations also experience coronary microvascular abnormalities associated with endothelial dysfunction and microvascular remodeling [17]. In this study, most subjects were classified as non-obese, with a median BMI of 26.97 (24.01–30.89), which could explain the difference in findings.

The coronary artery diameter, on the other hand, significantly predicts CSF (3.99 ± 0.53 vs. 3.41 ± 0.51, p < 0.001) in this study. Even after controlling for RBS and creatinine levels, the coronary artery diameter remained a significant predictor (adjusted OR 10.08, 95% CI 2.64–38.50, p < 0.001), with an optimal cut-off point of more than 3.56 mm with a sensitivity of 76.7% and specificity of 70.7% (AUC = 0.787, p < 0.001). This finding challenges the commonly held assumption that larger coronary diameters result in reduced probabilities of myocardial ischemia. It suggests that there exists a critical threshold beyond which coronary arteries, when exceeding a certain diameter, may detrimentally impact myocardial perfusion. Yang et al.'s publication shows that mean coronary artery diameter is also a significant predictor both in bivariate analysis (5.50 ± 0.85 mm vs. 5.18 ± 0.91 mm, p < 0.001) and in multivariate logistic regression analysis (adjusted OR 2.64, 95% CI 1.54–4.51, p < 0.001) [11].

The occurrence of CSF is seen in larger coronary artery diameters, according to the laws of physics, which state that the larger the radius of the blood vessels, the slower the speed of blood flow. This is calculated by the formula Q = πr2v, where Q is constant traffic, π is a constant of 3.14, r is the radius, and v is the flow velocity [11]. However, there are variations in the location of coronary arteries involved. In this study, most CSF cases were in the LCx (83.3%), while most publications report that LAD is the most commonly affected coronary artery [3, 5, 18]. LAD is a much longer vessel than LCx and RCA, which explains why CSF is more common in LAD [10]. This study found that LCx has greater tortuosity than LAD and RCA, affecting coronary blood flow. This explanation is in line with Mihic et al.'s publication, which states that tortuosity is a significant predictor (p < 0.001) in patients with non-obstructive ischemic symptoms, and LCx is the most tortuous vessel [19].

According to this study, RBS was found to be the second most significant predictor of CSF (105.5 (97.0–135.0) vs. 97.5 (95.0–115.0), p = 0.049). However, when multivariate logistic regression analysis was conducted, RBS was no longer significant (p = 0.066). Studies have shown that blood sugar levels, as determined by the HbA1C examination, can potentiate other predictors. Elsanan et al. published that in subjects with an HbA1C > 7, the NLR (r = 0.548, p < 0.001), Hb (r = 0.382, p = 0.018), and hematocrit (r = 0.542, p < 0.001) became significant predictors [13]. Hyperglycemia conditions have been shown to disrupt the physiology of blood flow. Kersten et al. published that hyperglycemia significantly disrupts coronary collateral blood flow through nitric oxide (NO)-mediated mechanisms [20]. The findings were reinforced by Angeli et al., who mentioned that hyperglycemia interferes with NO activation and increases the production of reactive oxygen species, worsening coronary blood flow in ACS cases [21].

Blood viscosity is an essential factor that affects blood flow, with hematocrit and plasma being the primary determinants. The characteristics of red blood cells (RBC) mainly determine microcirculation blood flow, so any deformities in RBC can increase blood viscosity. Therefore, parameters such as RDW are also crucial in determining the occurrence of CSF [22]. Platelet aggregation has been shown to increase significantly in people with CSF, so the platelet size presented by MPV becomes a critical marker describing platelet activity [12]. MPV is a biomarker of platelet activity that is very useful and easy to examine. MPV was also found to be a strong and independent predictor of impaired reperfusion and 6-month mortality in ST-segment elevation myocardial infarction patients undergoing PCI, as well as the incidence of restenosis and acute stent thrombosis [23].

Certain inflammatory predictors, like PLR and NLR, are known to be related to various inflammatory diseases, including cardiovascular disease because inflammation triggers endothelial dysfunction [6, 13]. An increased PLR level can even impact the prothrombotic status, slowing down the coronary blood flow [6]. High PLR levels are associated with a higher risk of recurrence of myocardial infarction, stroke, heart failure, and no-reflow syndrome after PCI [23]. Renal dysfunction also increases the risk of cardiovascular events and worsens prognosis. It is still associated with the mechanism of endothelial dysfunction and worsening of the atherosclerosis process caused by elevated creatinine levels [6]. Endothelial dysfunction affects the decrease in nitric oxide (NO) bioactivity, directly impacting the coronary microvascular [12].

The normal values for creatinine levels, RDW, PDW, and MPV vary depending on the laboratory's examination tools. This study's normal range for creatinine levels was 0.9–1.3 mg/dL, RDW 35.0–47.0 fL, PDW 9.0–13.0 fL, and MPV 7.2–11.1 fL. Several publications have indicated that these parameters significantly impact CSF. For creatinine levels, the results were 0.9 ± 0.2 [12] and 1.17 ± 0.23 [24], RDW 13.21 ± 1.76 [24], and MPV 13.10 ± 1.72 [24] in the CSF group. However, to date, there has been no publication on the effect of PDW on CSF. NLR and PLR are reliable indicators of systemic inflammation and have been extensively studied. However, there has been no consensus on the normal values of NLR and PLR as racial variations significantly influence them. For instance, a study on normal males and females in South China found the reference range for NLR to be 0.43–2.75 and 0.37–2.87, and for PLR to be 36.63–149.13 and 43.36–172.68, respectively [25]. Another publication reports that the normal NLR values in a healthy adult Belgian population are 0.78–3.53 [26]. Meanwhile, in the Iranian population with a mean sample age of 47.9 ± 9.29 years, the mean NLR and PLR were 1.70 ± 0.70 and 117.05 ± 47.73, respectively [27]. Several publications note the significant impact of NLR and PLR on CSF, with an NLR of 1.89 ± 0.58 [11] and a median PLR of 113.11 (91.13–140.11) [6]. Unfortunately, this study found that these parameters had no significant influence on CSF. The differences in results could be due to variations in the characteristics of the population studied in this research and the comparative study. The consistency and sample size, which originated from a single center, may have contributed to the disparity in results compared to the comparative study, which involved multiple centers and a larger number of subjects.

So far, the widely accepted pathophysiological approach for dealing with the CSF has been coronary microvascular dysfunction (CMD) and coronary endothelial dysfunction (CED). However, a surprising publication by Dutta et al. suggests that in patients with angina and non-obstructed coronary arteries, CSF and cTFC are not reliable indicators of CMD or CED. They propose that the guidelines supporting the use of cTFC in diagnosing CMD need to be reassessed. According to their findings, CSF had low diagnostic accuracy for both CMD (43.4%) and CED (31.7%), with poor sensitivity of 26.7% and 21.1%, respectively. Specificity was slightly higher at 65.2% for CMD and 56.0% for CED. Furthermore, cTFC could not predict CMD or CED, as indicated by ROC analyses with an AUC of 0.41 and 0.36, respectively [28]. Therefore, additional invasive or non-invasive tools are necessary to identify this clinical phenomenon when treating patients with CSF.

It is essential to note that although AF was an exclusion criterion in this study, there is a strong connection between AF and CSF. CAD and AF can exacerbate each other because they share similar risk factors and comorbidities [29, 30]. A study by Sharma et al. revealed that CSF was present in 42% of individuals with non-valvular AF. CSF can lead to myocardial ischemia even in the absence of obstructive CAD and may also increase hospitalization rates for AF patients due to fast ventricular response [29]. Furthermore, Gao et al. found that the incidence of CSF (adjusted OR 2.122, 95% CI 1.151–3.910, p = 0.016,) was significantly higher in the intraoperative AF episode group compared to the non-episode group. The proposed mechanism suggests that the duration of AF and the left atrial diameter can impact the TFC in AF patients. Additionally, acute AF leads to an increased demand for oxygen by the atria, potentially exceeding the oxygen supply. Moreover, a significant shortening of the diastolic phase can negatively affect diastolic-dominated coronary perfusion [30].

With the cause of CSF not fully understood, treatment options are limited. Administering anti-anginal medication only provides limited clinical benefits. Extensive studies testing pharmacological approaches to CSF are still lacking and existing evidence comes only from small studies with nonuniform inclusion criteria [7]. Empirical therapies based on several aspects include improving endothelial function by controlling cardiovascular risk factors, using nitrates to dilate coronary arteries, using beta-blockers to prolong coronary perfusion time, using antiplatelets to block platelet cross-linking and aggregation, and using calcium channel blockers to dilate coronary arteries and reduce myocardial contractility [6]. Physicians also widely use nicorandil, which has been proven to improve chest pain symptoms and the impaired function of the left ventricle. This improvement may be due to its potential to increase plasma NO and reduce endothelin-1 in CSF [31]. The effectiveness of nicorandil as a treatment is even better than that of nitroglycerin [32].

However, the study had several limitations. Even though data collection covered a span of seven years, the number of CSF subjects was relatively small. The study did not take into account biomolecular predictors that could have explained the mechanisms underlying CSF. Conducted in a single center with relatively homogeneous subjects, the results may not be easily generalized to the broader population. Furthermore, various echocardiography parameters, such as diastolic function, closely related to left ventricular end-diastolic pressure and CSF, could not be analyzed due to limited secondary data documentation. Lastly, many confounding variables, such as subject comorbidities and prior treatment, could not be controlled for.

## Conclusions

To summarize, the coronary artery diameter is a strong predictor of CSF in patients undergoing coronary angiography. Early medical intervention in patients with larger coronary artery diameters is expected to improve CSF outcomes significantly. However, more research is required with more subjects and multiple centers to confirm these findings.

## Data Availability

The datasets used and/or analyzed during the current study are available from the corresponding author on reasonable request. The case report form has been stored in the hospital file.

## Abbreviations

ACS: Acute coronary syndrome
AF: Atrial fibrillation
BMI: Body mass index
CAD: Coronary artery disease
CED: Coronary endothelial dysfunction
CMD: Coronary microvascular dysfunction
CSF: Coronary slow flow
cTFC: Corrected TIMI frame count
Hb: Hemoglobin
LAD: Left anterior descending artery
LCx: Left circumflex artery
MPV: Mean platelet volume
NECA: Normal epicardial coronary artery angiogram
NO: Nitric oxide
NLR: Neutrophil–lymphocyte ratio
PCI: Percutaneous coronary intervention
PDW: Platelet distribution width
PLR: Platelet-to-lymphocyte ratio
RBS: Random blood sugar
RCA: Right coronary artery
RDW: Red cell distribution width
TFC: TIMI frame count
